# Biological synthesis of coumarins in *Escherichia coli*

**DOI:** 10.1186/s12934-015-0248-y

**Published:** 2015-05-01

**Authors:** So-Mi Yang, Geun Young Shim, Bong-Gyu Kim, Joong-Hoon Ahn

**Affiliations:** Department of Bioscience and Biotechnology, Bio/Molecular Informatics Center, Konkuk University, 120 Neungdong-ro, Gwangjin-gu, Seoul, 143-701 Korea; Department of Forest Resources, Gyeongnam National University of Science and Technology, 33 Dongjin-ro, Jinju-si, Gyeongsangman-do, Jinju, 660-758 Korea

**Keywords:** Coumarins, Hydroxycinnamic acid, Feruloyl CoA 6′ hydroxylase, Metabolic engineering

## Abstract

**Background:**

Coumarins are a major group of plant secondary metabolites that serves as defense compounds against pathogens. Although coumarins can be obtained from diverse plant sources, the use of microorganisms to synthesize them could be an alternative way to supply building blocks for the synthesis of diverse coumarin derivatives.

**Results:**

Constructs harboring two genes, *F6′H* (encoding feruloyl CoA 6′ hydroxylase) and *4CL* (encoding 4-coumarate CoA:ligase), were manipulated to increase the productivity of coumarins. *Escherichia coli* expressing the two genes was cultured in medium supplemented with hydroxycinnamic acids (HCs) including *p*-coumaric acid, caffeic acid, and ferulic acid, resulting in the synthesis of the corresponding coumarins, umbelliferone, esculetin, and scopoletin. Cell concentration and initial substrate feeding concentration were optimized. In addition, umbelliferone, and esculetin were synthesized from glucose by using a *ybgC* deletion mutant and co-expressing tyrosine ammonia lyase and other genes involved in the tyrosine biosynthesis pathway.

**Conclusions:**

To produce coumarin derivatives (umbelliferone, scopoletin, and esculetin) in *E. coli*, several constructs containing *F6′H* and *4CL* were made, and their ability to synthesize coumarin derivatives was tested. The solubility of F6′H was critical for the final yield. After optimization, 82.9 mg/L of umbelliferone, 79.5 mg/L of scopoletin, and 52.3 mg/L of esculetin were biosynthesized from the corresponding HCs, respectively in *E. coli*. Umbelliferone and esculetin were also synthesized from glucose using engineered *E. coli* strains. The final yields of umbelliferone and esculetin were 66.1 and 61.4 mg/L, respectively.

## Background

Plants synthesize many types of phenolic compounds. Depending on their carbon skeletons, these phenolic compounds can be divided into four groups [1]. The first group is based on phenolic acids, whose carbon skeleton is C_6_-C_1_, and includes gallic acid, salicylic acid, and benzoic acid. The second group is hydroxycinnamic acids (HCs, C_6_-C_3_), which include *p*-coumaric acid, caffeic acid, and coumarin. The third group, the stilbenes, has a C_6_-C_2_-C_6_ skeleton, and includes resveratrol, piceatannol, and pallidol. The last group includes the flavonoids, which have a C_6_-C_3_-C_6_ skeleton, and includes quercetin, genistein, and apigenin. These plant phenolics are all synthesized from cinnamic acid derived from phenylalanine through the action of phenylalanine ammonia lyase (PAL). Malonyl-CoA supplies carbon to cinnamonyl-CoA to make the stilbenes and the flavonoids [2], while β-oxidation of cinnamic acid leads to the formation of phenolic acids [3].

Coumarins contain a backbone of 1,2-benzopyrone and are classified into four groups; simple coumarins, furanocoumarins, pyranocoumarins, and prenylated coumarins. Simple coumarins include scopoletin, umbelliferone, esculetin, and others [4]. Coumarins are plant secondary metabolites that are produced as either defensive compounds against pathogens [5,6] or iron chelators in the soil [7]. Biological activities of coumarins and their derivatives include antibacterial, antiviral, antifungal, anti-inflammatory, anticancer, anticoagulant, and antihypertensive activities [8]. As more impacts of coumarins on human have emerged, natural coumarins are serving as backbones for the synthesis of a range of new potentially useful coumarin derivatives [8,9].

**Figure 1:**
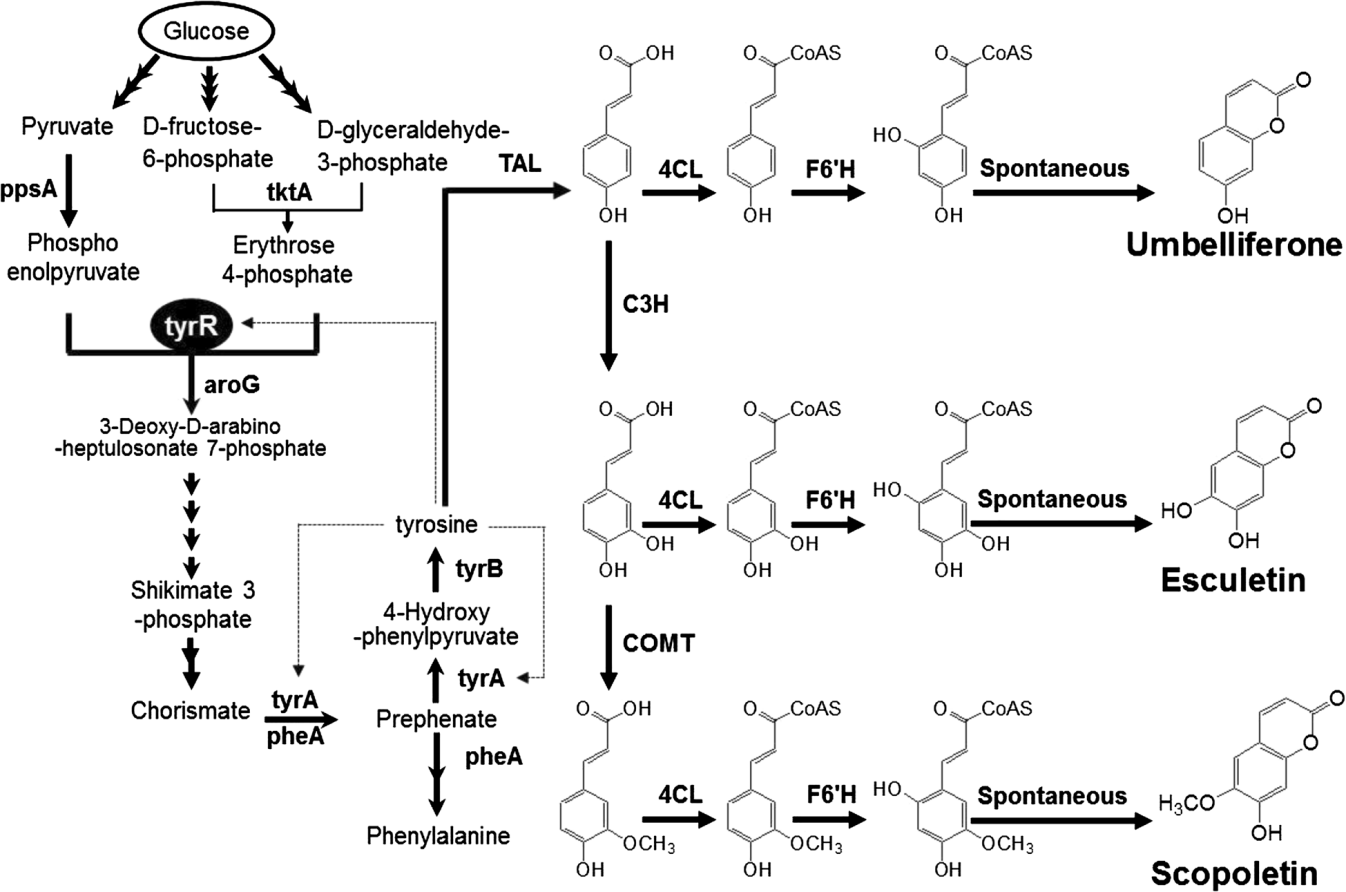
Biosynthesis pathway of coumarins starting with glucose. ppsA, phosphoenolpyruvate synthetase; tktA, transketolase; tyrR, phenylalanine DNA-binding transcription repressor; aroG, deoxyphosphoheptonate aldolase; tyrA, prephenate dehydrogenase; pheA, prephenate dehydratase; tyrB, phenylalanine aminotransferase; TAL, tyrosine amino lyase; 4CL, 4-coumaroyl-CoA ligase; C3H, coumarate 3-hydroxylase; COMT, caffeic acid *O*-methyltransferase; F6′H, feruloyl CoA 6′-hydroxylase. Tyrosine inhibits tyrR, and tyrA.

The biosynthetic pathway from cinnamic acid to coumarin was first elucidated in *Arabidopsis thaliana* [10]. The hydroxylation at the 6′-carbon of cinnamoyl-CoA by a 2-oxoglutarate-dependent dioxygenase (feruloyl CoA 6′-hydroxylase [F6′H], also known as *p*-coumaryol CoA 2′-hydroxylase [C2′H]) is a key step for the biosynthesis of coumarin [11]. In sweet potato (*Ipomoea batatas*), genes homologous to *F6*′*H* from *A. thaliana* (*IbF6′H1* and *IbF6′H2*) have been cloned and characterized [12]. These studies opened possibilities that three coumarins (umbelliferone, esculetin, and scopoletin) are synthesized from *p*-coumaric acid, caffeic acid, and ferulic acid, respectively, by a combination of *p*-cinnamic acid:CoA ligase (4CL) and F6′H [10,12] (Figure 1).

Model microorganisms have been used to synthesize plant phenolic compounds. Hydroxycinnamic acid, flavonoid, and stilbene have successfully been synthesized in *Escherichia coli* [13-15]. Coumarin can be synthesized from either phenylalanine or tyrosine using three enzymes, phenylalanine (or tyrosine) ammonia lyase (PAL or TAL), 4-cinnamic acid:coenzyme A ligase (4CL), and F6′H. Phenylalanine and tyrosine can be converted into cinnamic acid and *p*-coumaric acid by PAL and TAL, respectively [16,17]. Caffeic acid is synthesized from *p*-coumaric acid by hydroxylation using 4-hydroxyphenylacetate 3-hydroxylase (HpaBC) from *E. coli* [18] or a monooxygenase called Sam5 from *Saccharothrix espanaensis* [19]. Subsequent *O*-methylation of caffeic acid produces ferulic acid [20]. Attachment of coenzyme A to each HC and the spontaneous cyclization of 6′-hydroxycinnamoyl-CoAs in *E. coli* result in the formation of coumarins. Simple coumarins and their derivatives have been synthesized from either hydroxycinnamic acid or glucose [21-23]. This study used *F6′H* from *A. thaliana* and *I. batatas* and *4CL* from *A. thaliana* to produce umbelliferone and scopoletin from *p*-coumaric acid and ferulic acid, respectively, In addition, by employing *TAL*, *HapBC*, and *CCoAOMT* (caffeoyl CoA *O*-methyltransferase) along with *F6′H* and *4CL*, Lin et al. [21] also synthesized umbelliferone and scopoletin. But, the final yield of coumarins synthesized by this method was low. The bottleneck for the synthesis of coumarin is probably the conversion of hydroxycinnamoyl-CoA to 6′-hydroxy cinnamoyl-CoA. In this paper, we overcome this block by increasing the solubility of F6′H, thereby increasing the yield of coumarin. We report here the biosynthesis of coumarins from both hydroxycinnamic acids and glucose.

## Results

### Production of coumarin from hydroxycinnamic acid in *E. coli*

**Figure 2:**
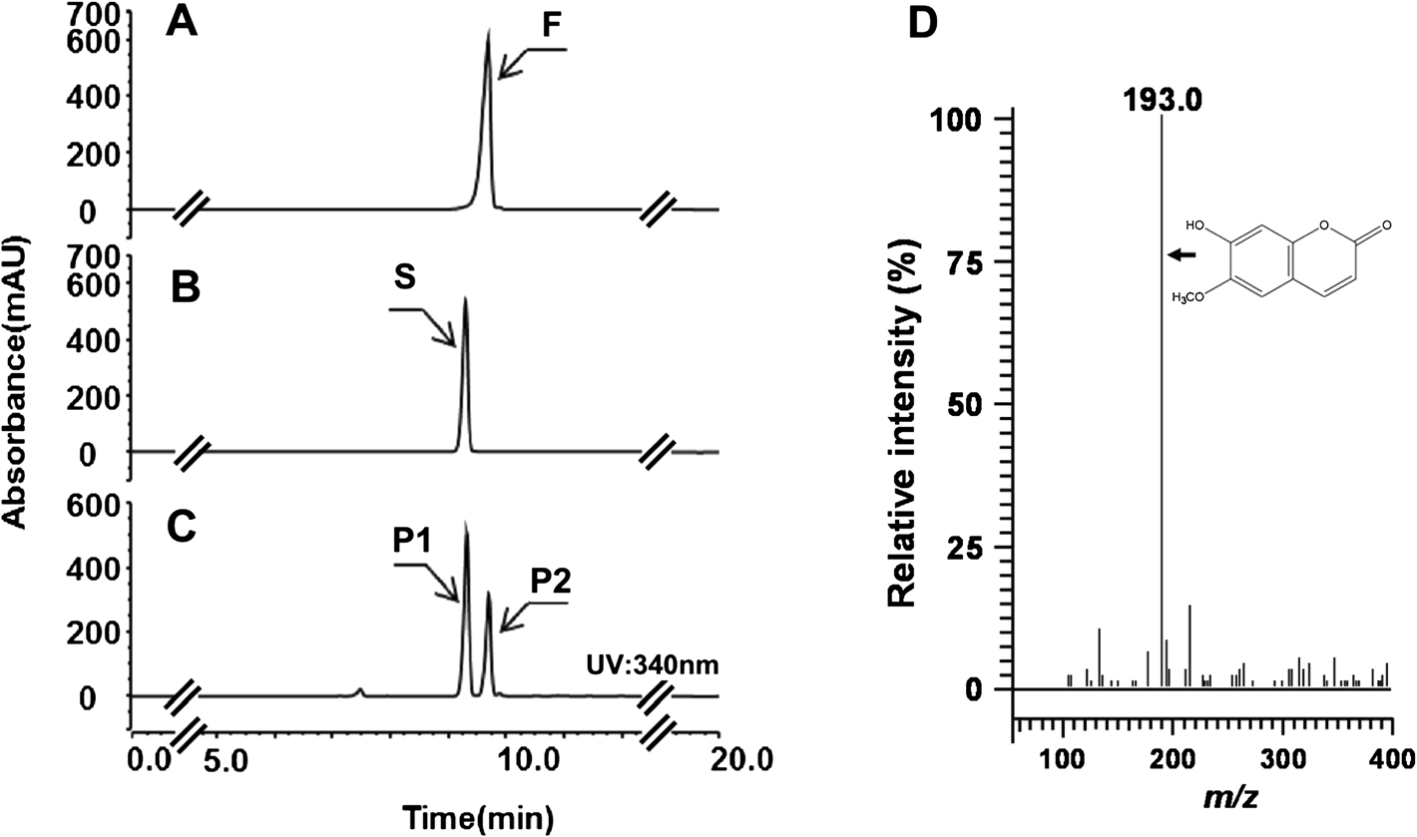
Production of scopoletin by feeding ferulic acid to *E. coli* strain B-CM1, **A**: ferulic acid standard (F), **B**: scopoletin standard (S), **C**: reaction products, P1 and P2, **D**: Mass spectrum of reaction product P1.

Coumarin is synthesized from hydroxycinnamic acid by the products of two genes, *4CL* and *F6′H*. Genes from *Oryza sativa* (*Os4CL*) and *I. batatas* (*IbF6′H2*) were cloned and transformed into *E. coli*. The *E. coli* transformant (B-CM1 in Table 1) was fed with ferulic acid. The reaction product was analyzed using high performance liquid chromatography (HPLC). As shown in Figure 2C, the culture filtrate from B-CM1 showed a new peak that had the same retention time as a standard of scopoletin. The molecular mass of the reaction product was 192-Da (g/mol) (Figure 2D), which corresponds to the predicted molecular mass of scopoletin. B-CM1 produced 3.5 mg/L scopoletin.

**Table 1:** **Plasmids and strains used in the present study**

**Plasmids or** ***E. coli*** **strain**	**Relevant properties or genetic marker**	**Source or reference**
Plasmids		
pACYCDuet	P15A ori, Cm^r^	Novagen
pCDFDuet	CloDE13 ori, Str^r^	Novagen
pETDuet	f1 ori, Amp^r^	Novagen
pGEX 5X-3	pBR322ori, Amp^r^	GE Healthcare
pA-SeTAL	pACYCDuet carrying *TAL* from *Saccharothrix espanaensis*	Kim et al. (2013) [26]
pA-aorG-SeTAL-tyrA	pACYCDuet carrying *TAL* from *S. espanaensis, aroG* and *tyrA* from *E. coli*	Kim et al. (2013) [26]
pA-aorG^fbr^-SeTAL-tyrA^fbr^	pACYCDuet carrying *TAL* from *S. espanaensis, aroG* ^fbr^ *,* and *tyrA* ^fbr^ from *E. coli*	This study
pA-aroG^fbr^-ppsA-tktA-SeTAL-tyrA^fbr^	pACYCDuet carrying *TAL* from *S. espanaensis, aroG* ^fbr^ *, PPSA, tktA,* and *tyrA* ^fbr^ from *E. coli*	Kim et al. (2013) [26]
pE-pIbF6′H2-pOs4CL	pETDuet harboring *F6′H2* from *Ipomoea batatas* and *4CL* from *Oryza sativa*. Each gene is controlled by independent T7 promoter.	This study
pE-pIbF6′H2-Os4CL	pETDuet harboring *F6′H2* from *Ipomoea batatas* and *4CL* from *Oryza sativa*. Each gene is controlled by one T7 promoter.	This study
pG-pIbF6′H2-pOs4CL	pGEX 5X-3 harboring *F6′H2* from *Ipomoea batatas* and *4CL* from *Oryza sativa. F6′H2* was fused with glutathione *S*-transferase. *F6′H2* is controlled by pTac promoter and *4CL* is controlled by T7 promoter.	This study
pG-pIbF6′H2-Os4CL	pGEX 5X-3 harboring *F6′H2* from *Ipomoea batatas* and *4CL* from *Oryza sativa. F6′H2* was fused with glutathione *S*-transferase. Each gene is controlled by one pTac promoter.	This study
pG-pIbF6′H1-Os4CL	pGEX 5X-3 harboring *F6′H1* from *Ipomoea batatas* and *4CL* from *Oryza sativa. F6′H2* was fused with glutathione *S*-transferase. Each gene is controlled by one pTac promoter.	This study
BL21 (DE3)	F^-^ *ompT hsdS*_*B*_(r_B_^-^ m_B_^-^) *gal dcm lon* (DE3)	Novagen
BtyrR	BL21(DE3) *ΔtyrR*	Kim et al. (2013) [26]
BtyrR-tyrA	BL21(DE3) *ΔtyrR/*Δ*tyrA*	Kim et al. (2013) [26]
BydiI	BL21(DE3) *ΔydiI*	This study
BybgC	BL21(DE3) *ΔybgC*	This study
B-CM1	BL21 harboring pE-pIbF6′H2-Os4CL	This study
B-CM2	BL21 harboring pE-pIbF6′H2-pOs4CL	This study
B-CM3	BL21 harboring pG-pIbF6′H2-pOs4CL	This study
B-CM4	BL21 harboring pG-pIbF6′H2-Os4CL	This study
B-CM5	BL21 harboring pE-pIbF6′H1-Os4CL	
B-CM6	BL21 harboring pG-pIbF6′H2-Os4CL and pA-SeTAL	This study
B-CM7	BL21 harboring pG-pIbF6′H2-Os4CL and pA-aroG-SeTAL-tyrA	This study
B-CM8	BL21 harboring pG-pIbF6′H2-Os4CL and pA-aroG^fbr^-SeTAL-tyrA^fbr^	This study
B-CM9	BL21 harboring pG-pIbF6′H2-Os4CL and pA-aroG^fbr^-ppsA-tktA-SeTAL-tyrA^fbr^	This study
B-CM10	BydiI harboring pG-pIbF6′H2-Os4CL and pA-aroG-SeTAL-tyrA	This study
B-CM11	BybgC harboring pG-pIbF6′H2-Os4CL and pA-aroG-SeTAL-tyrA	This study
B-CM12	BybgC harboring pG-pIbF6′H1-Os4CL, pA-aroG^fbr^-SeTAL-tyrA^fbr^, and pC-Sam5	This study

**Figure 3:**
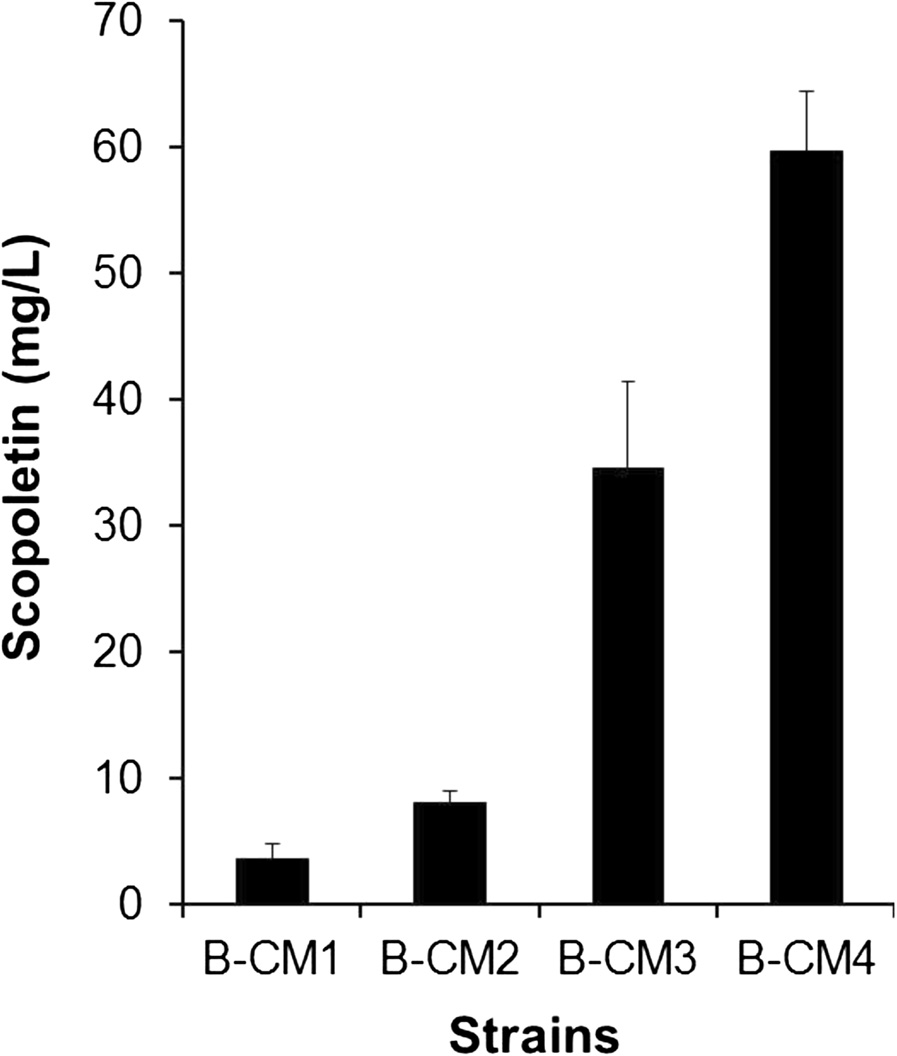
Comparison of different constructs for the production of scopoletin. B-CM1: BL21 harboring pE-pIbF6′H2-Os4CL, B-CM2: BL21 harboring pE-pIbF6′H2-pOs4CL, B-CM3: BL21 harboring pG-pIbF6′H2-pOs4CL, B-CM4: BL21 harboring pG-pIbF6′H2-Os4CL.

F6′H is a dioxygenase. Dioxygenases are sometimes poorly expressed in *E. coli* due to low solubility. Therefore, we fused F6′Hs to glutathione *S*-transferase (GST) to increase the solubility and stability. GST-fusion proteins have been shown to exhibit increased solubility and stability compared to His-tagged fusion proteins [24]. In addition, we made operon-type and pseudo operon-type constructs with *4CL* and *F6′H*. The operon-type construct has one T7 promoter which controls the expression of both *4CL* and *F6′H*. On the other hand, each gene is controlled by an independent T7 promoter in the pseudo operon-type construct. Therefore, we generated four constructs and tested them for the production of scopoletin from ferulic acid (B-CM1 to B-CM4). In two constructs, pG-pIbF6′H2-pOs4CL and pG-pIbF6′H2-Os4CL (Table 1), *IbF6′H2* was fused with *GST*. Two genes, *IbF6′H2* and *Os4CL*, were controlled by either two promoters in pG-pIbF6′H2-pOs4CL and pE-pIbF6′H2-pOs4CL (pseudo-operon type) or one promoter in pG-pIbF6′H2-Os4CL and pE-pIbF6′H2-Os4CL (operon type). In cases in which one promoter controlled two genes, a ribosome binding site (RBS) was attached in front of each gene. In addition, *IbF6′H* was located before *Os4CL* when both genes were controlled by one promoter. This orientation accumulated a fewer reaction intermediates [25]. Each construct was transformed into *E. coli* BL21 (DE3) and the resulting strains were tested for the ability to produce scopoletin. The strains harboring *IbF6′H2* fused with *GST* showed more scopoletin production than strains harboring *IbF6′H2* alone. B-CM3 and B-CM4, both of which contained the GST-IbF6′H2 fusion protein, produced approximately 34.6 and 59.7 mg/L of scopoletin, respectively, whereas B-CM1 and B-CM2 produced 3.5 and 8.1 mg/L, respectively (Figure 3). This indicates that the GST-fusion resulted in a higher yield of scopoletin, presumably due to increased solubilization and stability of IbF6′H2. Therefore, we decided to use the operon-type construct fused with GST (pG-pIbF6′H2-Os4CL).

Using strain B-CM4, the effect of the medium on the production of scopoletin was examined. After protein induction, cells were resuspended in LB, M9, or YM9 (M9 containing 1% yeast extract), and incubated with ferulic acid. Cells grown in LB accumulated more scopoletin than the other media (59.2 mg/L scopoletin vs. 13.1 mg/L in YM9 and 4.3 mg/L in M9).

**Figure 4:**
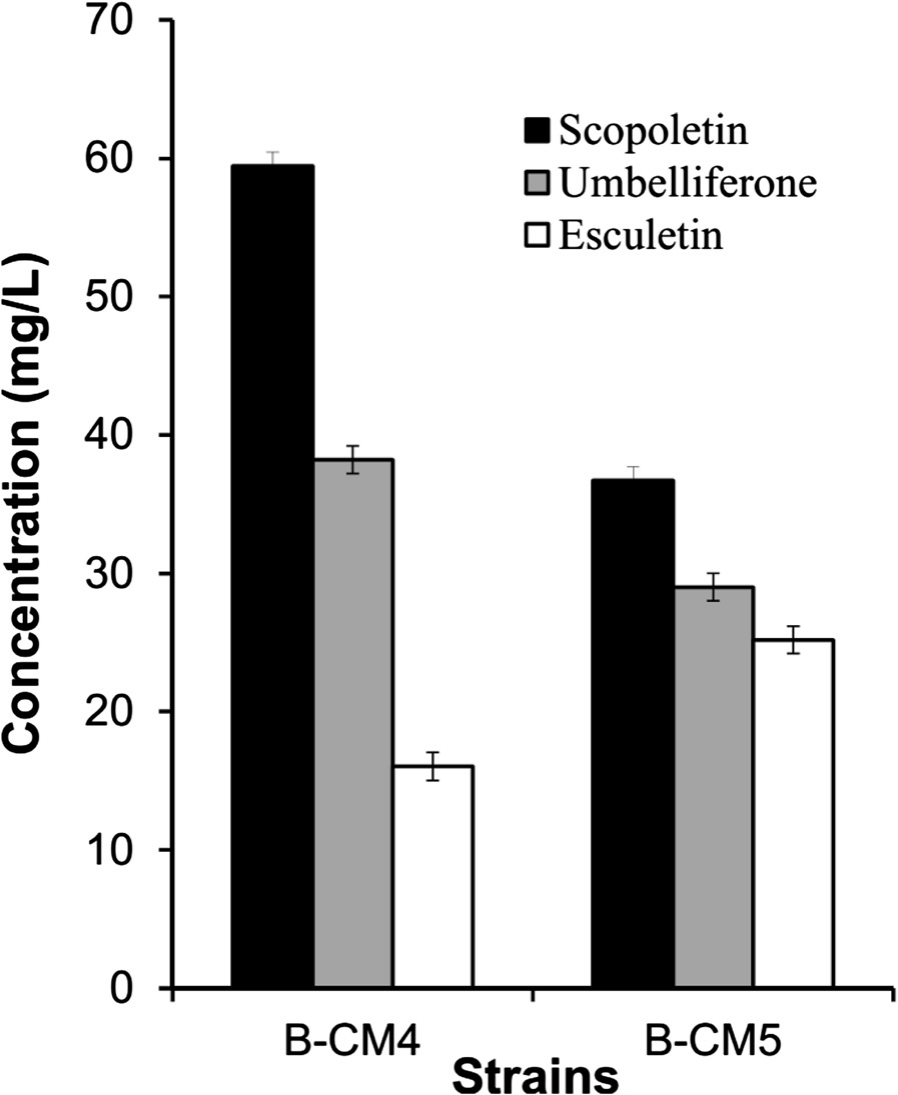
Substrate preference of F6′H1 and F6′H2 Strain B-CM4 contains F6′H2 while B-CM5 contains F6′H1.

We also generated a *GST-IbF6′H1* fusion gene, which was subcloned with *Os4CL* in an operon-type configuration (pG-pIbF6′H1-Os4CL). *E. coli* strains B-CM4, which contains pG-pIbF6′H2-Os4CL, and B-CM5, which contains pG-pIbF6′H1-Os4CL, were tested for production of umbelliferone, esculetin, and scopoletin from *p*-coumaric acid, caffeic acid, and ferulic acid, respectively. The two F6′Hs are expected to exhibit different substrate specificity. All *E. coli* strains converted *p*-coumaric acid, caffeic acid, and ferulic acid into umbelliferone, esculetin, and scopoletin, respectively. The structures of the products were confirmed using nuclear magnetic resonance (NMR) spectroscopy (see Materials and methods). However, the yield was different depending on the *E. coli* strain used and the substrate. B-CM4 produced about 38.2 mg/L umbelliferone, but B-CM5 produced 29.0 mg/L. Scopoletin was also produced at a higher amount in B-CM4 (approximately 59.4 mg/L) than in B-CM5 (33.6 mg/L). On the other hand, more esculetin was produced in B-CM5 (approximately 25.2 mg/L) than in B-CM4 (16.0 mg/L) (Figure 4). IbF6′H2 was superior for the production of umbelliferone and scopoletin, while IbF6′H1 was superior for the production of esculetin.

### Optimization of umbelliferone, scopoletin, and esculetin production

The optimal initial concentration of substrates (*p*-coumaric acid, caffeic acid, and ferulic acid) was examined for the production of umbelliferone, scopoletin, and esculetin, respectively. B-CM4 was used for the production of umbelliferone and scopoletin while B-CM5 was used for the production of esculetin. Approximately 90% of the *p*-coumaric acid was converted to umbelliferone at 0.3, 0.4, 0.5 mM *p*-coumaric acid. The production of umbelliferone continued to increase until 0.7 mM *p*-coumaric acid, after which the yield was not further increased very much, and *p*-coumaric acid accumulated. Therefore, 0.7 mM of *p*-coumaric acid was selected as the initial substrate concentration. Next, we determined the optimal cell density at 0.7 mM *p*-coumaric acid. Production of umbelliferone continued to increase until OD_600_ = 5, after which the yield decreased. B-CM4 at a cell density at OD_600_ = 5, produced approximately 0.51 mM (82.9 mg/L) of umbelliferone from 0.7 mM (114.9 mg/L) *p*-coumaric acid for a conversion yield of approximately 73%.

Using the same approach, the optimal initial substrate concentration and cell density were determined for both the production of scopoletin from ferulic acid and the production of esculetin from caffeic acid. The optimal substrate concentration and cell density for both scopoletin and esculetin were 0.7 mM at OD_600_ = 5, respectively. Under these conditions, approximately 79.5 mg/L of scopoletin (0.41 mM; 59% conversion) and 52.3 mg/L (0.29 mM; 41% conversion) of esculetin were produced after a 12 h reaction.

### Synthesis of umbelliferone and esculetin from glucose

**Figure 5:**
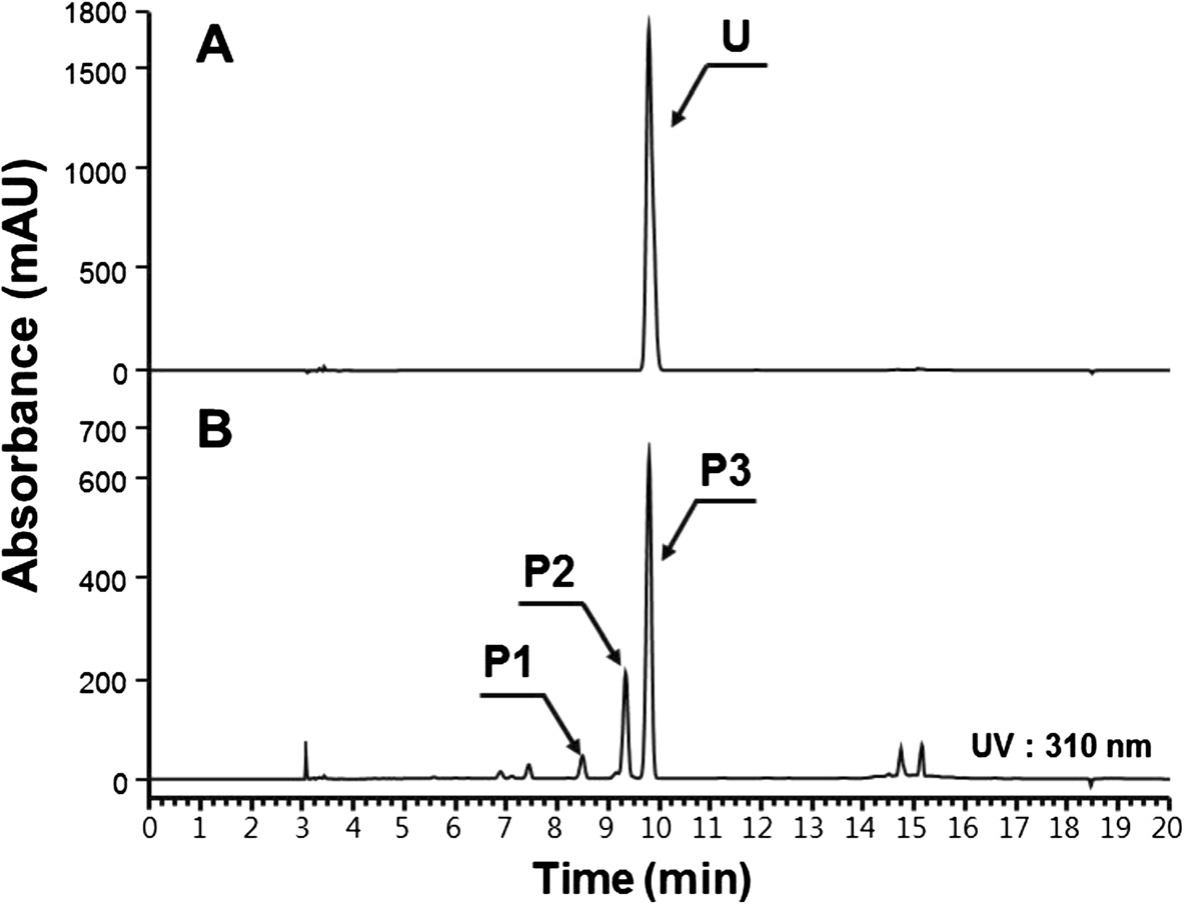
Production of umbelliferone from glucose using B-CM6. **A**, standard umbelliferone; **B**, reaction product from B-CM6 (P1, 2-hydroxy *p*-coumaric acid; P2, *p*-coumaric acid; P3, umbelliferone).

Umbelliferone and esculetin are synthesized from *p*-coumaric acid, and caffeic acid, respectively. *p*-Coumaric acid is synthesized from tyrosine by the action of tyrosine ammonia lyase (TAL). Subsequently, 3′-hydroxylation of *p*-coumaric acid leads to the synthesis of caffeic acid. Therefore, for the synthesis of umbelliferone, an additional gene, *TAL* is needed, which was previously cloned in our lab [26]. *E. coli* BL21 (DE3) was transformed with the *TAL* gene along with pG-pIbF6′H2-Os4CL. The resulting strain, B-CM6, was used to synthesize umbelliferone. The analysis of the culture filtrate by HPLC showed a peak (peak 3 in Figure 5B) that has the same retention time as standard umbelliferone. The molecular mass of this reaction product was 162 Da (g/mol), which is the predicted molecular mass of umbelliferone (data not shown). Therefore, umbelliferone was successfully synthesized by strain B-CM6.

**Figure 6:**
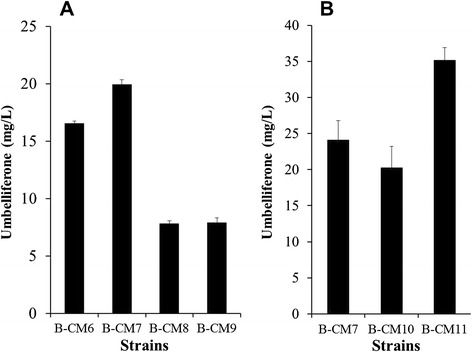
Analysis of reaction product from B-CM12. **A**, standard esculetin; **B**, reaction product from B-CM12 (P1, esculetin; P2, caffeic acid; P3, *p*-coumaric acid; P4, umbelliferone).

*p*-Coumaric acid is synthesized from tyrosine. Therefore, the intracellular tyrosine concentration is likely to have an effect on the yield of umbelliferone. In order to increase tyrosine production in *E. coli*, different combinations of four genes (*aroG*, *tyrA*, *ppsA*, and *tktA*) were used. These four genes have been used previously [26]. B-CM7, in which *aroG* and *tyrA* were overexpressed, produced more umbelliferone than other strains (23 mg/L) (Figure 6). The strains B-CM8 and B-CM9, in which feedback-free *aroG* (*aroG*^*fbr*^) and *tyrA* (*tyrA*^*fbr*^) [27] were overexpressed, produced less umbelliferone than the strain B-CM6, in which neither *aroG* nor *tyrA* was overexpressed. Although it is known that the feedback-free version of aroG and tyrA increase the production of tyrosine [28], this result indicates that there might be a certain optimal concentration of tyrosine beyond which umbelliferone yield does not increase, and even decrease probably due to the metabolic load of producing too much tyrosine.

During the synthesis of umbelliferone, two thioester intermediates (*p*-coumaroyl-CoA and 6′-hydroxy *p*-coumaroyl-CoA) are synthesized. These two intermediates might be degraded by thioesterase(s). To prevent the degradation of the two thioester intermediates and thereby increase the final yield of umbelliferone, two thioesterase mutants (BydiI and BybgC in Table 1) were made, and the ability of each mutant to produce umbelliferone was tested. ydiI has esterase activity toward 1,4-dihydroxy-2-naphthoyl-coenzyme A [29]. *YbgC* was predicted to encode acyl-CoA thioesterase. B-CM10 (Table 1) derived from BydiI, produced 17.2 mg/L umbelliferone, which was less than B-CM7 derived from *E. coli* BL21 (DE3) (23.2 mg/L). On the other hand, B-CM11 derived from BybgC synthesized approximately 32.1 mg/L umbelliferone (Figure 6), indicating that ybgC may hydrolyze the thioester bond in *p*-coumaroyl-CoA and/or 2-hydroxy *p*-coumaroyl-CoA. Therefore, we decided to use the strain BybgC to produce umbelliferone.

**Figure 7:**
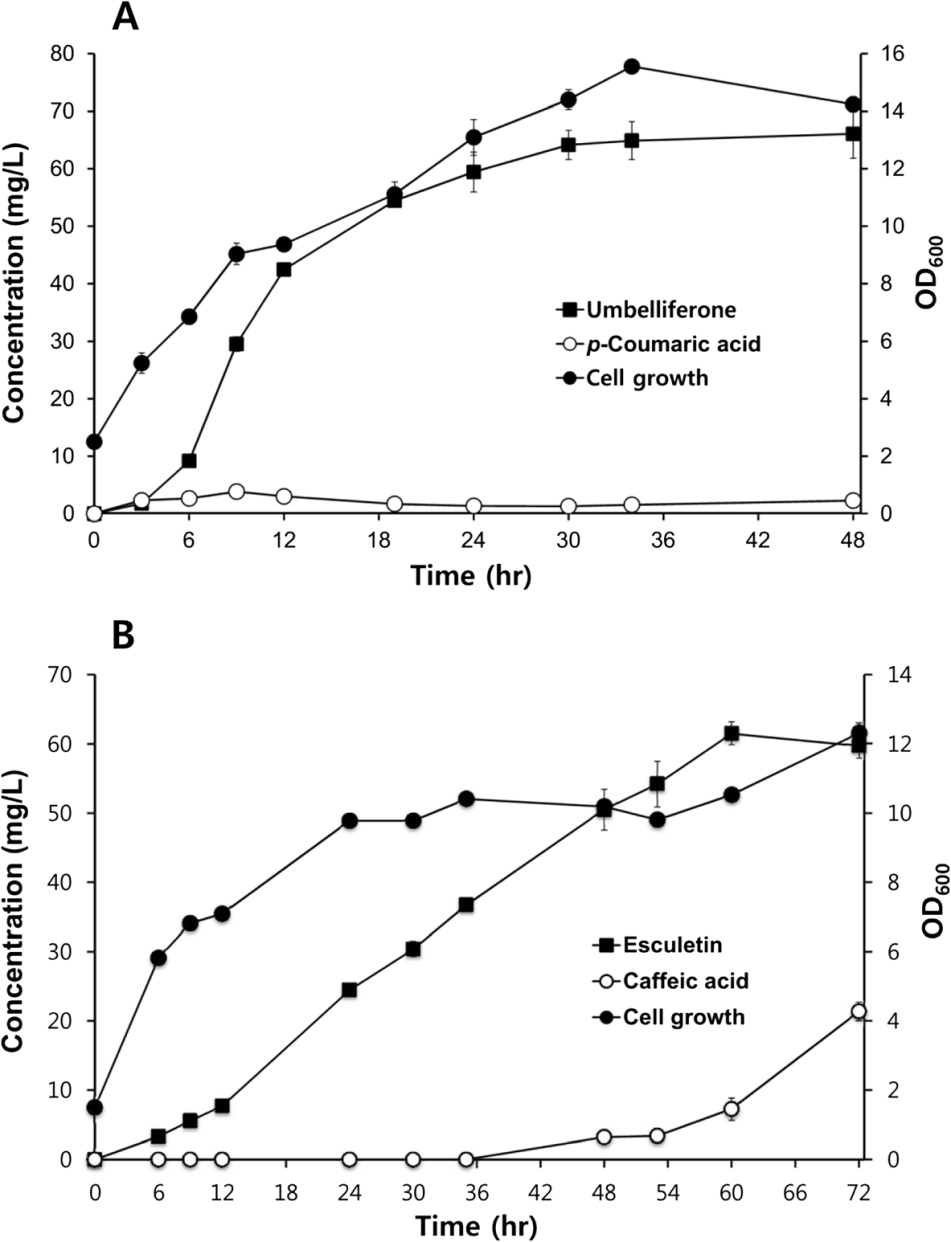
Effect of constructs **(A)** and *E. coli* mutant **(B)** on the production of umbelliferone from glucose.

Strain B-CM11 was used to produce umbelliferone from glucose. First, the optimal B-CM11 cell density for umbelliferone production was examined. Cell density was adjusted from OD_600_ = 0.5 to 3.0. The yield of umbelliferone increased from 32.2 mg/L at OD_600_ = 0.5 to 67.2 mg/L at OD_600_ = 2.5, but at OD_600_ = 3.0, it decreased to 48.4 mg/L. Therefore, a cell density of OD_600_ = 2.5 was used as the cell density for umbelliferone production. Next, the incubation temperature of B-CM11 was examined. B-CM11 was grown at 20, 25, 30, and 37°C. The highest production of umbelliferone occurred in cells grown at 25°C (68.2 mg/L). At a lower or higher temperature, umbelliferone production dramatically decreased. The yields at 20°C and 30°C were approximately 36.3 mg/L and 22.5 mg/L, respectively. Umbelliferone at 37°C was one-tenth the productivity at 30°C. The different yields at different temperatures may relate to the growth rate of cells and the expression of the introduced genes. Low temperature lowers the rate of cell growth, while high temperature can influence the expression of the introduced genes. Therefore, the optimal temperature is the one that does not hinder cell growth and maximizes expression of the introduced gene.

Using strain B-CM11, umbelliferone production was monitored for 48 h. Initial cell concentration was OD_600_ = 2.5, and the cells were grown at 25°C. Umbelliferone was synthesized rapidly from 6 to 18 h, after which production was not increased dramatically. The production reached a maximum at 36 h. The final yield was 66.1 mg/L (Figure 7A), which was less than that obtained by feeding *p*-coumaric acid (82.9 mg/L). Although B-CM11 continued to synthesize *p*-coumaric acid, most of it was converted into umbelliferone. This indicated that the overall metabolic flow from the production of *p*-coumaric acid to the production of umbelliferone is well-balanced.

**Figure 8:**
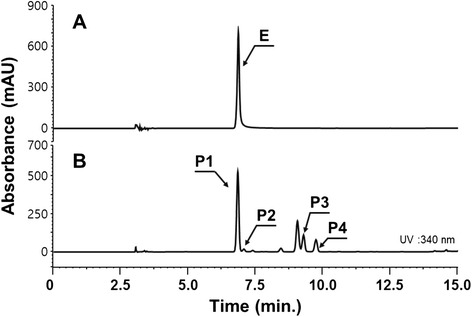
Production of umbelliferone **(A)** and esculetin **(B)** using B-CM11 and B-CM12 from glucose, respectively.

Esculetin was also synthesized from glucose by adding one additional gene (*Sam5*) that encodes a protein to convert *p*-coumaric acid into caffeic acid. We also tested the four constructs (pA-SeTAL, pA-aroG-SeTAL-tyrA, pA-aroG^fbr^-SeTAL-tyrA^fbr^, and pA-aroG^fbr^-ppsA-tktA-SeTAL-tyrA^fbr^) for the production of esculetin. The strain harboring pA-aorG^fbr^-SeTAL-tyrA^fbr^ produced more esculetin than other strains harboring different constructs (data not shown). Therefore, B-CM12 was used for the production of esculetin. The product was confirmed based on HPLC retention time and the MS/MS fragmentation pattern (Figure 8 and data not shown). The optimal cell concentration was determined to be OD_600_ = 1.5, and the optimal incubation temperature was 25°C. Using the optimized conditions, production of esculetin was monitored. Esculetin production needs more time than umbelliferone because of an additional step for the conversion of *p*-coumaric acid into caffeic acid. Esculetin production continued to increase until 60 h, at which point approximately 61.4 mg/L esculetin was synthesized (Figure 7B), similar to the amount which was obtained from feeding caffeic acid. At 60 h, the production of esculetin was maximum and caffeic acid began to accumulate.

## Discussion

Three coumarins, umbelliferone, esculetin, and scopoletin were synthesized by feeding the corresponding hydroxycinnamic acids to *E. coli* harboring *4CL* and *F6′H*. The yields ranged from 52.3 mg/L for esculetin, 79.5 mg/L for scopoletin, and 82.9 mg/L for umbelliferone. The final yields of these three coumarins were much higher than previous reported, 4.3 and 27. 8 mg/L for umbelliferone and scopoletin, respectively [21], although the catalytic efficiency of the F6′H used in the previous study was better than that used in the current study [10,12]. There are several possible explanations for the difference in the final yields. First, the tagged system of F6′H seems to be critical. The soluble form of F6′H was critical for the final yield. The GST-fusion of F6′H is more soluble form of F6′H. In addition, conversion of each HC into the corresponding HC-CoA seems to be an important point, because the 4CL, used in the two studies were different. Second, expression of *4CL* and *F6′H* was affected by the number of promoters. The pseudo-type construct of *4CL* and *F6′H*, in which expression of the two genes was controlled by two independent T7 promoter, gave less scopoletin than the operon-type, in which the expression of both genes was controlled by one T7 promoter. It was also shown that *E. coli* harboring the operon-type construct of *4CL* and *CHS* (chalcone synthase) synthesized more pinocembrin than *E. coli* harboring the pseudo-operon type [30]. The order of genes in the construct seemed to be important. Lin et al. [21] used the operon-type construct with *4CL* and *F6′H*. However, *F6′H* was place in front of *4CL* in their construct, which is opposite of ours. Although it was possible that the use of different *4CL* genes in the two studies could have contributed to the difference in the final yield, the order of genes in the construct could be another factor that influenced the final yield. The gene that acts at the later stage of a metabolic pathway should be placed in front of the gene that acts at the earlier stage in order to accumulate less intermediate. It was observed that the gene order in the construct influences the final yield of the product [25,30].

Umbelliferone and esculetin were also synthesized from glucose. It was surprising that the final yield from glucose was comparable to that from the feeding study. It is generally known that final yield is decreased when more genes are added to *E. coli*. The use of an *E. coli ybgC* deletion mutant might prevent the degradation of HC-CoA, which is the substrate of F6′H. The use of *E. coli ybgC* deletion mutant resulted in higher production of esculetin and umbelliferone from glucose. However, deleting *ybgC* did not have any effect on the production of esculetin and umbelliferone when caffeic acid and *p*-coumaric acid were fed to *E. coli*. It seems that the conversion of caffeoyl-CoA or *p*-coumaroyl-CoA into esculetin or umbelliferone is a bottle neck for the overall reaction. When either caffeic acid or *p*-coumaric acid was fed to *E. coli*, the corresponding CoA derivatives were formed by 4CL, which is faster than conversion of them into esculetin or umbelliferone by F6′H, and the amount of caffeoyl-CoA or *p*-coumaroyl-CoA was dependent only on the amount of caffeic acid or *p*-coumaric acid, respectively. Therefore, although some caffeoyl-CoA or *p*-coumaroyl-CoA is degraded by ybgC, caffeoyl-CoA or *p*-coumaroyl-CoA are still available for the next reaction catalyzed by F6′H. On the other hand, when caffeic acid or *p*-coumaric acid are synthesized from tyrosine and then converted into the corresponding CoAs, most of the caffeoyl-CoA or *p*-coumaroyl-CoA was degraded by ybgC and the remaining amounts of them were not enough for the capability of F6′H, and this therefore resulted in decreasing the production of esculetin or umbelliferone. Therefore, deletion of ybgC in *E. coli* prevents degradation of caffeoyl-CoA or *p*-coumaroyl-CoA and increases the final yields. We cannot exclude the possibility that ybgC degrades 6′-hydroxy caffeoyl-CoA or 6′-hydroxy *p*-coumaroyl-CoA.

## Conclusions

Coumarins are plant secondary metabolites that contain a backbone of 1,2-benzopyrone. Natural coumarins serve as a backbone for the synthesis of more active derivatives. We used *E. coli* to synthesize coumarins. *E. coli* strains harboring the optimized construct of *F6′H* and *4CL* were used to synthesize umbelliferone, esculetin, and scopoletin from *p*-coumaric acid, caffeic acid, and ferulic acid, respectively. Umbelliferone (82. 9 mg/L), scopoletin (79.5 mg/L), and esculetin (52.3 mg/L) were synthesized after the optimization of cell concentration and the initial substrate feeding concentration. In addition, umbelliferone, and esculetin were synthesized from glucose. A *ybgC* deletion mutant (BydgC), which was assumed to prevent the degradation of either hydroxycinnamoyl-CoA or 6′-hydroxy hydroxycinnamoyl-CoA was used and this strain was transformed with *TAL* and other genes involved in the tyrosine biosynthesis pathway. Using these strategies, we produced 66.1 mg/L of umbelliferone, and 61.4 mg/L of esculetin.

## Materials and methods

### Constructs

*4CL* (4-coumarate: CoA ligase) from *Oryza sativa* was cloned previously [31]. *p*-Coumaroyl Coenzyme A/feruloyl Coenzyme A *ortho*-hydroxylases from sweet potato (*Ipomoea batatas: IbF6′H1* [GenBank ID: AB636153] and *IbF6′H2* [GenBank ID: AB636154]) were cloned using reverse transcription-polymerase chain reaction (RT-PCR). Total RNA from sweet potato tuber tissue was isolated using Plant Total RNA Isolation Kit (Qiagen, Hilden, Germany), and cDNA was synthesized with Omniscript reverse transcript (Qiagen). The forward primers of *IbF6′H1* and *IbF6′H2* were 5′-ATGGCTCCAACACTCTTGAC-3′ and 5′-ATGAATCAAACACTCGCTGC-3′, respectively. The reverse primers were 5′-TCAGATCTTGGCGTAATCGA-3′ and 5′-TCAAATGTTGGCAAAATCGA-3′. The resulting PCR product of each gene was subcloned into pGEM-T easy vector (Promega, Madison, USA) and sequenced. Each gene was then reamplified with the forward primer containing an *Eco*RI site, and the reverse primers containing a *Not*I site. The PCR product was subcloned into the *Eco*RI/*Not*I sites of pET-Duet (Novagen). *Os4CL*, which was cloned previously [31], was amplified using PCR with 5′-AT*CATATG*GGGTCGGTGGCGGCGGAGGAGG-3′ and 5′-AT*CTCGAG*TTAGCTGCTTTTGGGCGCATC-3′ (*Nde*I site and *Xho*I site are indicated as italic), and subcloned into the *Nde*I/*Xho*I sites of pET-Duet1 containing *IbF6′H1* or *IbF6′H2*. Each gene in these constructs was controlled by an independent T7 promoter. The plasmids were called pE-pIbF6′H1-pOs4CL or pE-pIbF6′H2-pOs4CL (Table 1). To make a construct in which both genes were controlled by one promoter (operon-type), *Os4CL* was amplified with a forward primer containing a ribosomal binding site (RBS) and a *Not*I site (5′-AT*GCGGCCGC*aaggagatataccaATGGGGTCGGTGGCG-3′; *Not*I site is indicated as italic and RBS is shown in lower case), and the reverse primer containing a *Xho*I site (5′-AT*CTCGAG*TTAGCTGCTTTTGGGCGCATC-3′; *Xho*I site is indicated as italic). The resulting PCR product was subcloned into the *Not*I/*Xho*I sites of pET-Duet1 containing *IbF6′H1* or *IbF6′H2*. The resulting constructs, pE-pIbF6′H1-Os4CL and pE-pIbF6′H2-Os4CL contained a single promoter but an RBS site in front of each gene. pE-pIbF6′H1-Os4CL, and pE-pIbF6′H2-Os4CL were digested with *Eco*RI and *Xho*I, and the fragment containing *IbF6′H1* and *Os4CL* or *IbF6′H2* and *Os4CL* was subcloned into the *Eco*RI/*Xho*I sites of pGEX 5X-3 (GE Healthcare, USA). The resulting constructs were named pG-pIbF6′H1-Os4CL, and pG-pIbF6′H2-Os4CL, respectively. To make the construct with two promoters and *F6′H* fused with *GST*, PCR was carried out using pE-pIbF6′H1-Os4CL, or pE-pIbF6′H2-Os4CL as a template using Pfu DNA polymerase with the primers, 5′-AT*GAATTC*GATGCCTTCAACAACACTCTCC-3′ for *IbF6′H1* (EcoRI site is indicated as italic) or 5′-AT*GAATTC*GATGATGCCTTCAACAACACTC-3′ for *IbF6′H2* (*Eco*RI site indicated as italic) and 5′-TTAGCTGCTTTTGGGCGCATC-3′ for Os4CL. The resulting PCR product was digested with EcoRI and subcloned into the EcoRI/SmaI sites of pGEX 5X-3. The resulting constructs were pG-pIbF6′H1-pOs4CL and pG-pIbF6′H2-pOs4CL, respectively.

The *TAL* gene from *Saccharothrix espanaensis* (SeTAL), *aroG*, *tyrA*, and the feedback-free versions of aroG (*aroG*^*fbr*^) and tyrA (*tyrA*^*fbr*^) were cloned previously [26]. For the pA-aroG^fbr^-SeTAL-tyrA^fbr^ construct, aroG^fbr^ and tyrA^fbr^ were introduced into the *Eco*RI/*Sal*I and the *Nde*I/*Kpn*I sites of pACYCDuet, respectively, and was named pA-aroG^fbr^-tyrA^fbr^. The SeTAL gene was cloned into the *Eco*RI/*Not*I sites of pACYDUet and named pA-SeTAL. *SeTAL* containing the T7 promoter and RBS was amplified with two primers flanking *Xho*I and *Not*I using pA-SeTAL as a template. The PCR product was digested with *Xho*I/*Not*I and ligated into the corresponding sites of pA-aroG^fbr^-tyrA^fbr^. The resulting construct was named pA-aroG^fbr^-SeTAL-tyrA^fbr^. pA-aroG-SeTAL-tyrA was constructed using the same method described above.

### Production of coumarins in *E. coli*

*E. coli* transformants containing pG-IbF6′H1-Os4CL or pG-IbF6′H2-Os4CL were grown in LB containing 50 μg/mL ampicillin for 16 h at 37°C. This culture was inoculated into fresh LB containing 50 μg/mL ampicillin and grown to an OD_600_ = 0.8. Isopropyl β-D-1-thiogalactopyranoside (IPTG) was added at a final concentration of 1 mM and the culture was grown for another 6 h at 25°C. Cells were harvested, and the cell concentration was adjusted to OD_600_ = 3 with 10 mL of fresh LB containing 50 μg/mL ampicillin in 100 mL flask. The substrate (*p*-coumaric acid, caffeic acid, or ferulic acid) was added at a concentration of 400 μM. The resulting culture was incubated at 30°C for 12 h with shaking. The effect of medium on the production of scopoletin was examined using LB, M9 (containing 2% glucose), or YM9 (M9 containing 2% glucose and 0.2% yeast extract). We used 10 mL of each medium in 100 mL flask.

To determine the substrate concentration to produce the highest yield of coumarin derivatives, substrate was added at 0.3, 0.4, 0.5, 0.7, 0.9, 1.2, or 1.5 mM. The cell density was OD_600_ = 3. The mixture was incubated at 30°C for 12 h with shaking at 180 rpm. For the optimal cell density, cell density was adjusted to OD_600_ = 1, 2, 3, 5, or 10. Substrate was added at 0.7 mM, and the mixture was incubated at 30°C for 12 h with shaking at 180 rpm. The reaction scale was used as described above.

The culture (200 μL) was extracted twice with the equal volume of ethylacetate. The organic phase was recovered and evaporated to dryness. The remaining residue was dissolved in 60 μL of dimethylsulfoxide (DMSO) and analyzed using a Thermo Ultimate 3000 HPLC equipped with a photo diode array (PDA) detector and a Varian C18 reversed-phase column (Varian, 4.60 × 250 mm, 3.5 μm particle size) by injecting 10 μL. The mobile phases consisted of 0.1% formic acid in water or acetonitrile. The program was: 15% acetonitrile at 0 min, 35% acetonitrile at 10 min, 90% acetonitrile at 12 min, 90% acetonitrile at 15 min, 15% acetonitrile at 15.1 min, and 15% acetonitrile at 20 min. The flow rate was 1 mL/min, and the separation was monitored at 290, 310, and 340 nm.

Umbelliferone, scopoletin, and esculetin were purchased from Sigma (St. Louis, MO, USA) and used as standards to calculate yields of umbelliferone, scopoletin, or esculetin. The means and standard errors were calculated from triplicate experiments. Analysis of variance (ANOVA) was carried out using Tukey’s method with a significance level of P = 0.01 using 2010 Microsoft Office Excel.

Mass spectrometry (MS) was performed as described previously [31]. Structures of products were determined using nuclear magnetic resonance spectroscopy (NMR) [32]. The NMR data were as follows; Umbelliferone: ^1^H NMR (400 MHz, Acetone-*d*_*6*_) δ 7.87 (d, *J* = 9.5 Hz, 1H), 7.52 (d, *J* = 8.4 Hz, 1H), 6.85 (dd, *J* = 8.5, 2.1 Hz, 1H), 6.75 (d, *J* = 1.9 Hz, 1H), 6.17 (d, *J* = 9.5 Hz, 1H).

Scopoletin: ^1^H NMR (400 MHz, CDCl_3_) δ 7.52 (d, *J* = 9.4 Hz, 1H), 6.90 (s, 1H), 6.84 (s, 1H), 6.52 (d, *J* = 9.5 Hz, 1H), 3.88 (s, 3H).

Esculetin: ^1^H NMR (400 MHz, Acetone-*d*_*6*_) δ 7.79 (d, *J* = 9.6 Hz, 1H), 7.05 (s, 1H), 6.79 (s, 1H), 6.15 (d, *J* = 9.6 Hz, 1H).

### Synthesis of umbelliferone and esculetin from glucose

An overnight culture of each strain was inoculated into fresh LB medium containing 50 μg/mL of ampicillin, and chloramphenicol and the cells were grown at 37°C with shaking at 180 rpm until the OD_600_ exceeded 1.0. The cells were collected by centrifugation. The cell density was adjusted to an OD_600_ of 1.0 with 10 mL of M9 medium supplemented with 1% yeast extract, 2% glucose, 50 μg/mL of ampicillin and chloramphenicol and 1 mM IPTG in 100 mL flask and then incubated at 30°C for 48 h with shaking. The reaction product was analyzed by HPLC.

To determine the optimal cell concentration for the production of umbelliferone, B-CM11 cells were grown and protein expression was induced as described above. Cells were harvested by centrifugation and cell concentrations were adjusted to OD_600_ = 0.5, 1. 1.5, 2.0, 2.5, or 3.0 with M9 medium containing 2% glucose, 1% yeast extract, 50 μg/mL chloramphenicol and ampicillin, and 1 mM IPTG. Each strain was grown at 30°C for 48 h and production of umbelliferone determined by HPLC. To determine the optimum incubation temperature, the cell concentration of B-CM11 was adjusted to OD_600_ = 2.5, and cells were then incubated at 20, 25, 30, and 37°C for 48 h with shaking at 180 rpm. The yield of umbelliferone from each culture was determined by HPLC. Esculetin production from glucose using B-CM12 was carried out using similar methods to that of umbelliferone production.

## References

[1] Jaganath IB, Crozier A, Fraga CG) (2010). Dietary flavonoids and phenolic compounds. Plant Phenolics and Human Health: Biochemistry, Nutrition, and Pharmacology.

[2] Winkel-Shirley B (2001). Flavonoid biosynthesis. a colorful model for genetics, biochemistry, cell biology, and biotechnology. Plant Physiol.

[3] Qualley AV, Widhalm JR, Adebesin F, Kish CM, Dudareva N (2012). Completion of the core β-oxidation pathway of benzoic acid biosynthesis in plants. Proc Natl Acad Sci U S A.

[4] Bourgaud F, Hehn A, Larbat R, Doerper S, Gontier E, Kellner S (2006). Biosynthesis of coumarins in plants: a major pathway still to be unravelled for cytochrome P450 enzymes. Phytochem Rev.

[5] Chong J, Baltz R, Schmitt C, Beffa R, Fritig B, Saindrenan P (2002). Downregulation of a pathogen-responsive tobacco UDP-Glc:phenylpropanoid glucosyltransferase reduces scopoletin glucoside accumulation, enhances oxidative stress, and weakens virus resistance. Plant Cell.

[6] Sun H, Wang L, Zhang B, Ma J, Hettenhausen C, Cao G (2014). Scopoletin is a phytoalexin against *Alternaria alternata* in wild tobacco dependent on jasmonate signaling. J Exp Bot.

[7] Fourcroy P, Sisó-Terraza P, Sudre D, Savirón M, Reyt G, Gaymard F (2014). Involvement of the ABCG37 transporter in secretion of scopoletin and derivatives by Arabidopsis roots in response to iron deficiency. New Phytol.

[8] Venugopala KN, Rashmi V, Odhav B (2013). Review on natural coumarin lead compounds for their pharmacological activity. Biomed Res Int.

[9] Borges F, Roleira F, Milhazes N, Santana L, Uriarte E (2005). Simple coumarins and analogues in medicinal chemistry: occurrence, synthesis and biological activity. Curr Med Chem.

[10] Kai K, Mizutani M, Kawamura N, Yamamoto R, Tamai M, Yamaguchi H (2008). Scopoletin is biosynthesized via *ortho*-hydroxylation of feruloyl CoA by a 2-oxoglutarate-dependent dioxygenase in *Arabidopsis thaliana*. Plant J.

[11] Vialart G, Hehn A, Olry A, Ito K, Krieger C, Larbat R (2012). A 2-oxoglutarate-dependent dioxygenase from *Ruta graveolens* L. exhibits *p*-coumaroyl CoA 2′ hydroxylase activity (C2′H): a missing step in the synthesis of umbelliferone in plants. Plant J.

[12] Matsumoto S, Mizutani M, Sakata K, Shimizu B (2012). Molecular cloning and functional analysis of the *ortho*-hydroxylases of *p*-coumaroyl coenzyme a/feruloyl coenzyme a involved in formation of umbelliferone and scopoletin in sweet potato, *ipomoea batatas* (L.) Lam. Phytochemistry.

[13] Malla S, Koffas MAG, Kazlauskas RJ, Kim B-G (2012). Production of 7-*O*-methyl aromadendrin, a medicinally valuable flavonoid, in *Escherichia coli*. Appl Environ Microbiol.

[14] Miyahisa I, Funa N, Ohnishi Y, Martens S, Moriguchi T, Horinouchi S (2006). Combinatorial biosynthesis of flavones and flavonols in *Escherichia coli*. Appl Microbiol Biotehcnol.

[15] Watts KT, Lee PC, Schmidt-Dannert C (2006). Biosynthesis of plant-specific stilbene polyketides in metabolically engineered *Escherichia coli*. BMC Biotech.

[16] Cochrane FC, Davin LB, Lewis NG (2004). The *Arabidopsis* phenylalanine ammonia lyase gene family: kinetic characterization of the four PAL isoforms. Phytochemistry.

[17] Kyndt JA, Meyer TE, Cusanovich MA, Van Beeumen JJ (2002). Characterization of a bacterial tyrosine ammonia lyase, a biosynthetic enzyme for the photoactive yellow protein. FEBS Lett.

[18] Lin Y, Yan Y (2012). Biosynthesis of caffeic acid in *Escherichia coli* using its endogenous hydroxylase complex. Microb Cell Fact.

[19] Berner M, Krug D, Bihlmaier C, Vente A, Müller R, Bechthold A (2006). Genes and enzymes involved in caffeic acid biosynthesis in the actinomycete *Saccharothrix espanaensis*. J Bacteriol.

[20] Li L, Popko JL, Zhang X-H, Osakabe K, Tsai C-J, Joshi CP (1997). A novel multifunctional *O*-methyltransferase implicated in a dual methylation pathway associated with lignin biosynthesis in loblolly pine. Proc Natl Acad Sci U S A.

[21] Lin Y, Sun X, Yuan Q, Yan Y (2013). Combinatorial biosynthesis of plant-specific coumarins in bacteria. Met Eng.

[22] Lin Y, Shen X, Yuan Q, Yan Y (2013). Microbial biosynthesis of the anticoagulant precursor 4-hydroxycoumarin. Nat Commun.

[23] Lin Y, Yan Y (2014). Biotechnological production of plant-specific hydroxylated phenylpropanoids. Biotech Bioeng.

[24] Terpe K (2003). Overview of tag protein fusions: from molecular and biochemical fundamentals to commercial systems. Appl Microbiol Biotechnol.

[25] Lim CF, Fowler ZL, Hueller T, Schaffer S, Koffas MA (2011). High-yield resveratrol production in engineered *Escherichia coli*. App Environ Microbiol.

[26] Kim MJ, Kim B-G, Ahn J-H (2013). Biosynthesis of bioactive *O*-methylated flavonoids in *Escherichia coli*. Appl Microbiol Biotech.

[27] Lütke-Eversloh T, Stephanopoulos G (2007). *L*-Tyrosine production by deregulated strains of *Escherichia coli*. Appl Microbiol Biotechnol.

[28] Santos CNS, Koffas M, Stephanopoulos G (2011). Optimization of a heterologous pathway for the production of flavonoids from glucose. Met Eng.

[29] Chen M, Ma X, Chen X, Jiang M, Song H, Guo Z (2013). Identification of a hotdog fold thioesterase involved in the biosynthesis of menaquinone in *Escherichia coli*. J Bacteriol.

[30] Kim BG, Lee H, Ahn J-H (2014). Biosynthesis of pinocembrin from glucose using engineered *Escherichia coli*. J Microbiol Biotech.

[31] Lee Y-J, Jeon Y, Lee JS, Kim B-G, Lee CH, Ahn J-H (2007). Enzymatic synthesis of phenolic CoAs using 4-coumarate:coenzyme a ligase (4CL) from rice. Bull Korean Chem Soc.

[32] Yoon J-A, Kim B-G, Lee WJ, Lim Y, Chong Y, Ahn J-H (2012). (2012) Production of a novel quercetin glycoside through metabolic engineering of *Escherichia coli*. Appl Env Microbiol.

